# Pallidal lead placement in dystonia: leads of non-responders are contained within an anatomical range defined by responders

**DOI:** 10.1007/s00415-020-09753-z

**Published:** 2020-02-17

**Authors:** Simone Zittel, Ute Hidding, Maria Trumpfheller, Vanessa Lupici Baltzer, Alessandro Gulberti, Miriam Schaper, Maxine Biermann, Carsten Buhmann, Andreas K. Engel, Christian Gerloff, Manfred Westphal, Jana Stadler, Johannes A. Köppen, Monika Pötter-Nerger, Christian K. E. Moll, Wolfgang Hamel

**Affiliations:** 1 Department of Neurology, University Medical Center Hamburg-Eppendorf, Hamburg, Germany; 2 Brainlab AG, Munich, Germany; 3 Department of Neurophysiology and Pathophysiology, University Medical Center Hamburg-Eppendorf, Hamburg, Germany; 4 Department of Neurosurgery, University Medical Center Hamburg-Eppendorf, Hamburg, Germany

**Keywords:** Dystonia, GPi stimulation, Deep brain stimulation, Volume of tissue activated (VTA), Clinical outcome

## Abstract

**Background:**

Deep brain stimulation (DBS) within the pallidum represents an effective and well-established treatment for isolated dystonia. However, clinical outcome after surgery may be variable with limited response in 10–25% of patients. The effect of lead location on clinical improvement is still under debate.

**Objective:**

To identify stimulated brain regions associated with the most beneficial clinical outcome in dystonia patients.

**Methods:**

18 patients with cervical and generalized dystonia with chronic DBS of the internal pallidum were investigated. Patients were grouped according to their clinical improvement into responders, intermediate responders and non-responders. Magnetic resonance and computed tomography images were co-registered, and the volume of tissue activated (VTA) with respect to the pallidum of individual patients was analysed.

**Results:**

VTAs in responders (*n* = 11), intermediate responders (*n* = 3) and non-responders (*n* = 4) intersected with the posterior internal (GPi) and external (GPe) pallidum and the subpallidal area. VTA heat maps showed an almost complete overlap of VTAs of responders, intermediate and non-responders. VTA coverage of the GPi was not higher in responders. In contrast, VTAs of intermediate and non-responders covered the GPi to a significantly larger extent in the left hemisphere (*p* < 0.01).

**Conclusions:**

DBS of ventral parts of the posterior GPi, GPe and the adjacent subpallidal area containing pallidothalamic output projections resulted in favourable clinical effects. Of note, non-responders were also stimulated within the same area. This suggests that factors other than mere lead location (e.g., clinical phenotype, genetic background) have determined clinical outcome in the present cohort.

**Electronic supplementary material:**

The online version of this article (10.1007/s00415-020-09753-z) contains supplementary material, which is available to authorized users.

## Introduction

Pallidal deep brain stimulation (DBS) is an effective and well-established treatment for medical-refractory focal, segmental or generalized isolated dystonia [1, 2]. Responders to DBS treatment typically have a clinical improvement of more than 50% compared to the preoperative motor impairment but response may be variable [1–4]. Of note, 10–25% of isolated dystonia patients show insufficient benefit from surgery with clinical improvement less than 25–30% [3, 4]. So far, the reasons for different responses to globus pallidus internus (GPi) DBS are not completely understood. In particular, the definite causes for treatment failures are not known. Given possible complications and costs of this invasive therapy reliable outcome predictors of surgery are desirable. Several factors contribute to the postoperative outcome including disease duration, preoperative motor score, fixed skeletal deformities, genetic factors (*TOR1A, THAP1*), GPi volume, etiology of dystonia and lead location [5–7]. In a recent publication, it has been claimed that electrode misplacement may account for 50% of cases with poor treatment response [8]. However, retrospective analysis was restricted to dystonia patients who failed to respond to GPi stimulation, and it is unknown to what extent misplaced electrodes could be found in responders.

Although it is well established that electrode placement in the postero-ventro-lateral portion of the GPi, representing the sensorimotor part of the nucleus, is associated with good clinical outcome, the impact of variation in electrode location on clinical responses is hitherto unclear. Previous studies mainly focused on active contact localization [9–11] or mean electrical charge distribution [12] to define the optimal stimulation spot in dystonia patients. In the present retrospective single-center study, we investigated electrode location and the putative volume of tissue activated (VTA) according to a three-dimensional model in relation to treatment responses in a group of isolated dystonia patients.

## Patients and methods

Eighteen patients with isolated cervical (CD, *n* = 11) and generalized dystonia (GD, *n* = 7; three *TOR1A* and one *THAP1* gene mutation carrier) with chronic GPi DBS for at least 4 years were included into this retrospective study [13]. Patients with combined or complex dystonia were excluded from the study. Demographic data of the patients were collected including gender, age, disease duration at the time of surgery and follow-up interval after surgery. Quadripolar DBS electrodes (model 3389, Medtronic Inc., Minneapolis, MN, USA) were implanted in the postero-ventro-lateral GPi as reported previously [14]. Electrodes were later connected to a subcutaneously implanted impulse generator (Kinetra or Activa PC/RC, Medtronic).

According to a previous study motor symptoms were assessed with the Toronto Western Spasmodic Torticollis Rating Scale (TWSTRS) in CD patients and with the Burke Fahn Marsden Dystonia Rating Scale (BFMDRS) in GD patients [15]. Non-blinded motor ratings were based on standardized video recordings. Postoperative motor scores were given as calculated percentage change from the preoperative baseline value. According to the clinical response patients were grouped into responders (improvement > 50%), intermediate responders (improvement 25–50%) and non-responders (improvement < 25%) [1]. The study was performed according to the Declaration of Helsinki and approved by the local ethics committee. All subjects gave informed consent prior to inclusion in the study.

Individual coordinates for the active contacts relative to the midcommissural point were determined. The required anterior commissure-posterior commissure (ACPC)-system was defined manually.

**Fig. 1:**
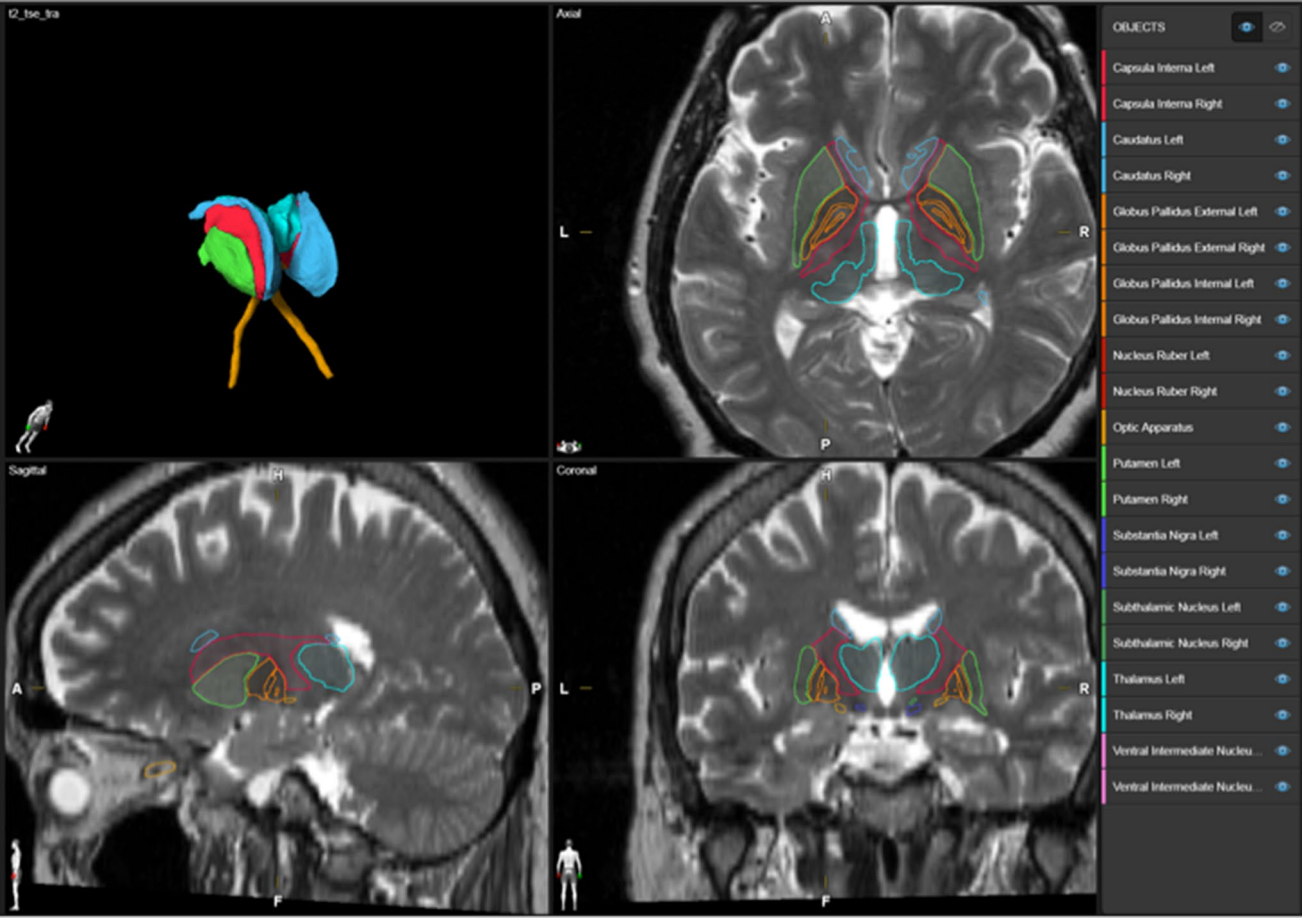
Three-dimensional auto-segmentation. Three-dimensional auto-segmentation of the basal ganglia by means of a proprietary algorithm (Brainlab, Munich, Germany). The different structures of the basal ganglia are indicated in different colors

Stimulation parameters at the last follow-up examination were used to calculate a three-dimensional stimulation field model that is assumed to represent the VTA. VTA modeling was based on algorithms developed by the McIntyre laboratory [16]. Parameters included amplitude, frequency, pulse width, impedance and active contacts. For patients stimulated in a constant voltage mode the applied current (mA) was calculated based on impedance and Ohm’s law. Magnetic resonance and computed tomography images of individual patients were co-registered. The leads were automatically detected with the ‘Lead Localization’ module and anatomical objects were created using the ‘Anatomical Mapping’ module of a commercially available software (Elements, Brainlab, Munich, Germany). Lead detection by the ‘Lead Localization’ module was verified by visual inspection. Three-dimensional reconstructions of all relevant basal ganglia nuclei and VTAs were created by the ‘Elements’ software (Brainlab, Munich, Germany) using a proprietary algorithm (Fig. 1).

Additionally, patient images were normalized to a common reference space (i.e., the Brainlab Atlas) by elastic multimodal image registration. Based on this registration all VTAs and leads were also transformed from the respective patient images to the common reference space where they were aggregated into a heat map.

For statistical analysis CD and GD patients were pooled due to the small sample size. Surgical targeting for CD and GD did not differ, and it is assumed that the optimal anatomical area for symptom alleviation in CD and GD is the same. The group of responders was compared with a merged group containing both intermediate and non-responders. Mean VTA volumes in cm^3^ ± standard deviation were calculated for each group. Additionally, VTA overlap with different anatomical structures (GPi, GPe and subpallidal area) was assessed and given in percentage of the total VTA. Two-sample *t* tests were applied to compare the VTA volume for each hemisphere between groups. Also, the GPi and GPe volume for each hemisphere was compared between groups with two-sample *t* tests. In a separate analysis, we evaluated the overlap of the VTA with different anatomical structures. The percentage of VTA coverage of the GPi, GPe and subpallidal area was analysed separately for each hemisphere with two-sample *t* tests between groups. Bonferroni correction was used to adjust for multiple comparisons. Correlation analyses were performed applying the Pearson`s correlation coefficient to investigate whether the VTA volume was associated with a lateral, anterior or inferior electrode location according to the AC-PC system. The influence of VTA volume or localization of active contacts on clinical outcome was evaluated. In addition, correlation analysis for demographic characteristics (age at the time of surgery, disease duration) and clinical outcome were performed (IBM SPSS Statistics, version 25.0). All statistical values are given as mean ± SD.

## Results

Eleven CD and seven GD patients were included into the analysis (13 female, 5 male patients). Mean age at the time of surgery was 45 ± 15 years. Mean follow-up interval after surgery was 9 ± 3 years. Disease duration at the time of surgery was 13 ± 9 years (Table 1). Average clinical improvement was 56.0 ± 30.6% in CD patients (TWSTRS) and 53.0 ± 45.8% in GD patients (BFMDRS). In CD patients, the proportion of intermediate responders was 27.3%. 18.2% of CD patients did not respond to GPi DBS as defined by our clinical criteria. In GD patients, the proportion of both intermediate and non-responders was 14.3% each.

**Table 1:** Individual demographic and clinical data of the patients are indicated

Patient	Age	Gender	Type of dystonia	Genetics	Disease duration at time of DBS	Follow-up after DBS	Clinical improvement (%)
1	50	M	CD	–	23	7	10
2	61	M	CD	–	5	4	− 5
3	49	M	CD	–	3	11	83
4	52	M	CD	–	4	6	33
5	68	F	CD	–	3	6	61
6	57	F	CD	–	5	6	37
7	52	F	CD	–	22	11	53
8	59	F	CD	–	28	10	50
9	60	F	CD	–	10	5	57
10	17	F	GD	*TOR1A*	7	10	53
11	36	F	GD	*TOR1A*	28	9	37
12	41	F	GD	–	6	9	− 56
13	21	F	GD	–	10	8	84
14	27	F	GD	*TOR1A*	17	9	55
15	49	F	CD	–	10	11	12
16	27	M	GD	*THAP1*	21	12	60
17	43	F	CD	–	29	12	94
18	39	F	GD	–	3	12	96

Mean stimulation parameters were: 3.8 ± 1.5 mA (3.4 ± 0.8 V), 123 ± 45 µs and 139 ± 38 Hz. None of the patients experienced stimulation-induced side effects that limited DBS programming.

The average coordinates for all active contacts relative to the midcommissural point were: *x* = 21.4 mm (± 1.5) lateral, *y* = 4.1 mm (± 1.8) anterior and *z* = − 3.3 mm (± 1.9) ventral. Of note, average stereotactic coordinates of patients responding to GPi DBS did not differ from the group of intermediate/non-responders: *x* = 21.4 vs. 21.3 mm; *y* = 4.1 vs. 4.1; *z* = − 3.6 vs. − 2.4, respectively.

Mean VTA volumes in responders were 0.34 ± 0.19 cm^3^ for the left hemisphere and 0.31 ± 0.16 cm^3^ for the right hemisphere. In intermediate/non-responders mean VTA volume was 0.21 ± 0.12 cm^3^ for the left hemisphere and 0.29 ± 0.27 cm^3^ for the right hemisphere. There was no significant difference in VTA volume between groups (left hemisphere *p* = 0.13, right hemisphere *p* = 0.89). Also, mean GPi (responders left 0.54 ± 0.09 cm^3^, right 0.51 ± 0.08 cm^3^, intermediate/non-responders left 0.60 ± 0.08 cm^3^, right 0.57 ± 0.08 cm^3^) and GPe (responders left 0.96 ± 0.14 cm^3^, right 0.96 ± 0.12 cm^3^, intermediate/non-responders left 0.99 ± 0.13 cm^3^, right 0.99 ± 0.15 cm^3^) volume was not different between groups.

**Fig. 2:**
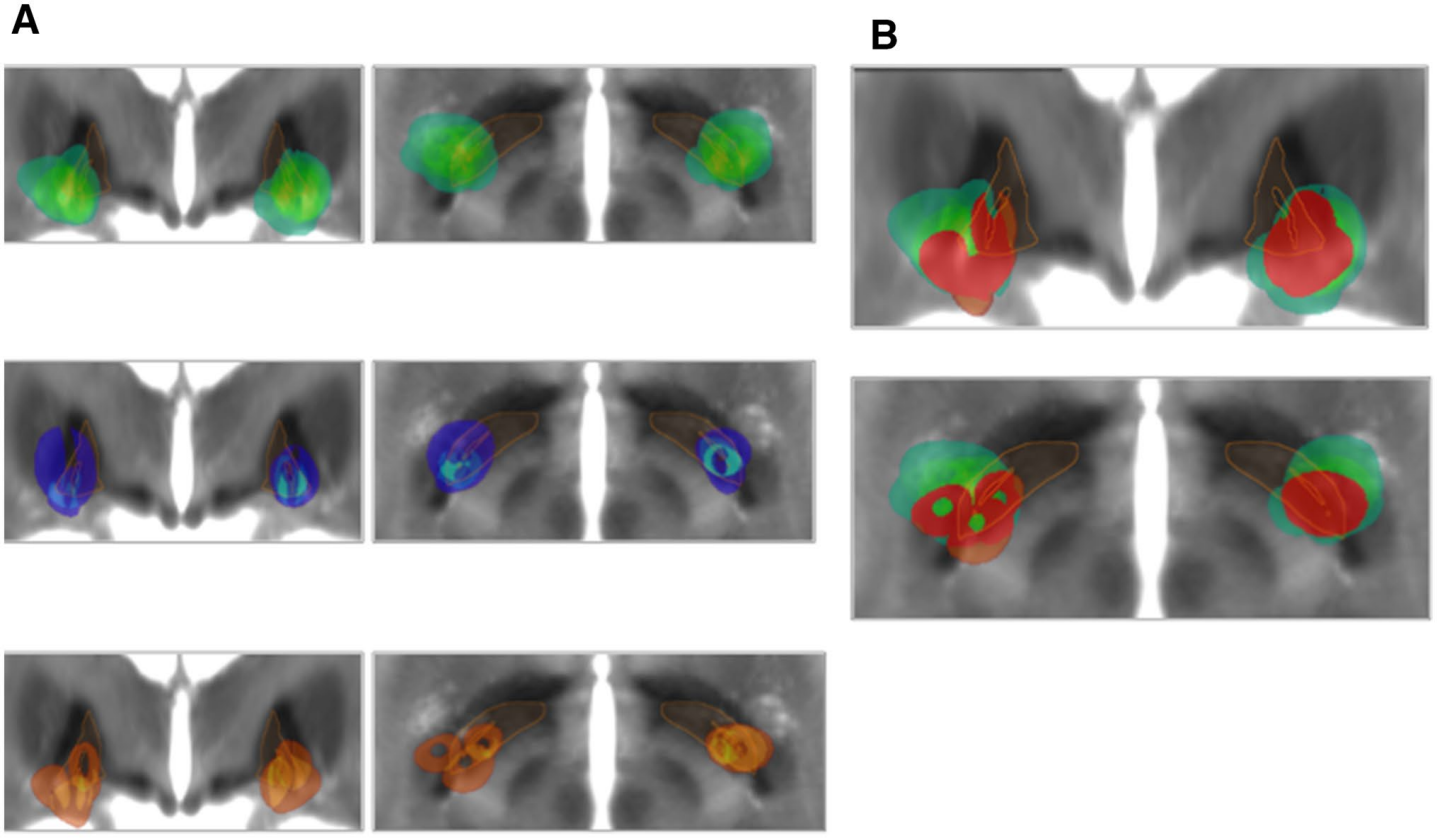
VTA heat maps. **a** VTAs are displayed on a normalized MRI which was created based on the Brainlab Atlas. VTA heat maps of responders (upper trace, green), intermediate responders (middle trace, blue) and non-responders (lower trace, orange) are displayed. The brightness of the colors indicates the degree of overlap between the VTAs of different electrodes. The GPi is indicated in yellow. Left images: coronal views, right images: axial views. **b** VTA heat maps of responders (green) vs. non-responders (red). Top row shows coronal views, bottom row axial views. The VTAs of non-responders are almost completely contained within the VTAs of responders

Heat maps consisting of aggregated VTAs of responders, intermediate responders and non-responders showed that the VTAs generally intersected with the posterior aspects of GPi, GPe and the subpallidal area, respectively (Fig. 2a). The internal capsule was practically excluded from the VTAs. Figure 2b demonstrates that the VTA heat maps of non-responders were contained within the more medial aspects of VTA heat maps observed for responders.

**Fig. 3:**
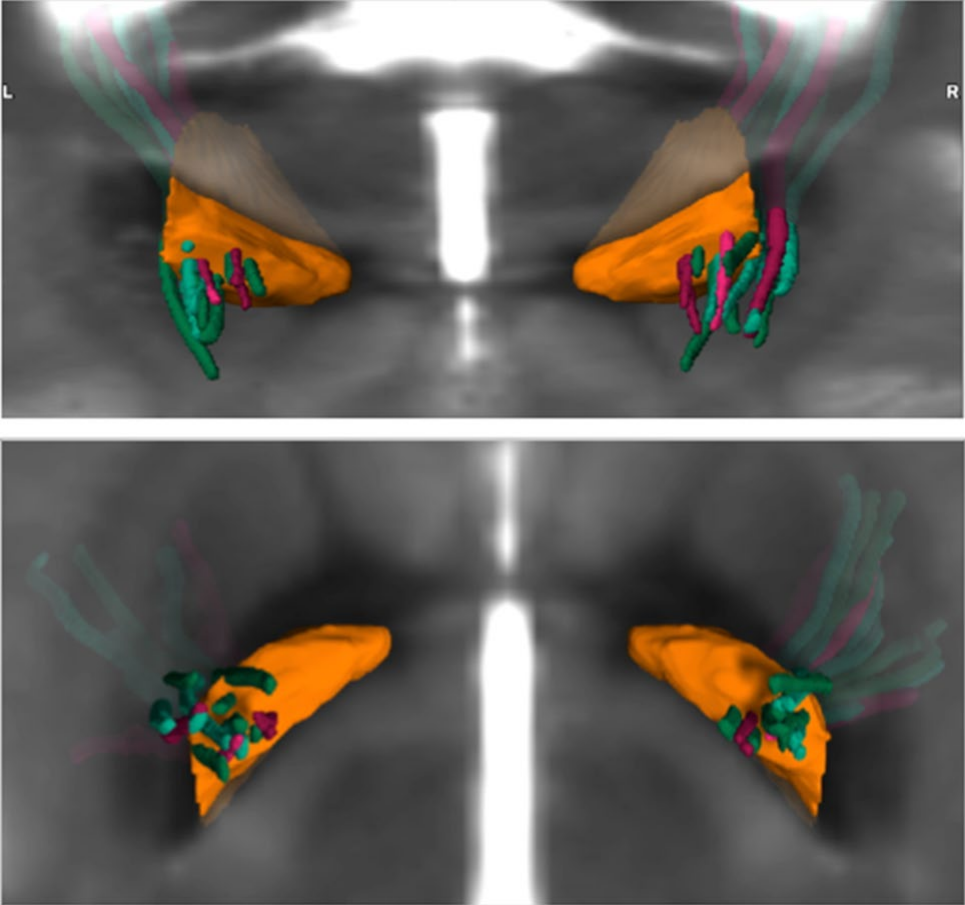
Three-dimensional imaging of electrodes. Three-dimensional imaging of electrodes of responders (green) and non-responders (red) with respect to the GPi (orange) of the Brainlab Atlas displayed on a normalized MRI. As the leads were transferred to the common reference space by elastic fusion, they are not straight but bent. There is no distinct clustering of electrodes of responders vs. non-responders

Furthermore, three-dimensional imaging of the electrodes by elastic multimodal image registration did not reveal clustering of electrodes or an obvious spatial pattern separating responders from non-responders (Fig. 3).

**Fig. 4:**
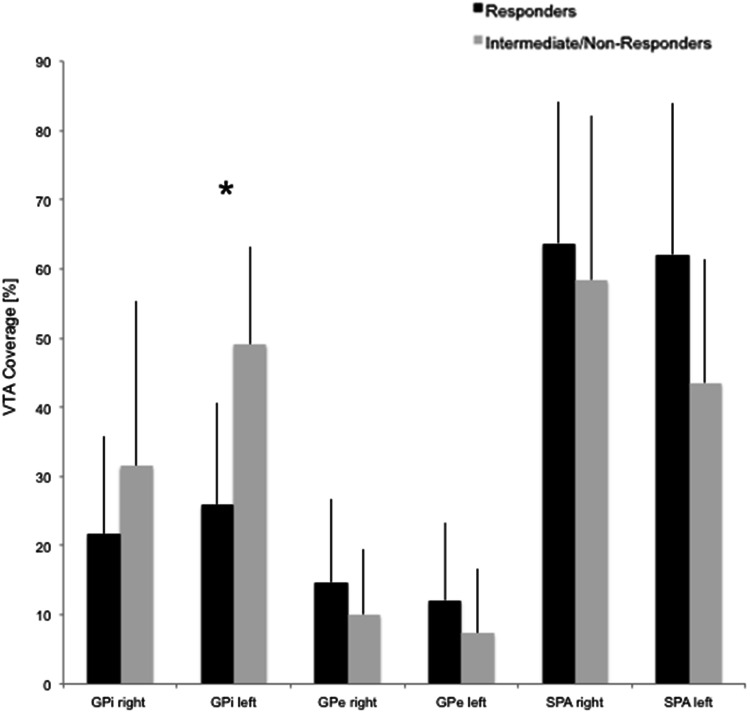
VTA coverage. The relative coverage (in percentage) of the Globus pallidus internus (GPi), Globus pallidus externus (GPe) and subpallidal area by VTAs of responders (black bars) and intermediate/non-responders (grey bars) is indicated. *Indicates a statistically significant difference (*p* < 0.05; *t* test with Bonferroni correction)

In the left hemisphere, the VTAs of responders covered significantly less of the GPi than observed for intermediate/non-responders (*p* < 0.01; Fig. 4). This difference, however, was not observed for the right hemisphere. There was a trend towards larger VTA coverage of the subpallidal area in the left hemisphere in responders compared to intermediate/non-responders, which did not reach the level of significance (*p* = 0.10). Supplement 1 displays individual VTAs of all patients in three-dimensional space and indicates that the VTAs of all but one patient extended into the subpallidal area.

Age at the time of surgery and disease duration were not correlated with clinical improvement after the operation. Also, clinical improvement did not correlate with lead location in any of the three directions in space or with VTA volume. However, the VTA size was correlated with lead location in the anterior direction (*r* = 0.48; *p* < 0.01), indicating that higher stimulation currents were used with more anteriorly located contacts. VTA size was not correlated with other directions in space.

## Discussion

The main finding of the present study is that the active electrode contacts of dystonia patients responding to pallidal stimulation or failing DBS therapy were found within the same anatomical range. Most electrodes were located in the postero-ventro-lateral GPi. This portion of the GPi is known to represent the sensorimotor territory, giving rise to projections to the basal ganglia recipient part of the motor thalamus [17]. In addition, the VTAs also covered parts of the GPe and typically extended largely into the subpallidal area.

In a previous study, smaller GPi volumes have been described in dystonia patients with a limited response to DBS [12]. This, however, has not been observed in our cohort in which the pallida of intermediate/non-responders tended to be larger than those of responders without significant difference.

Similar to our findings, in a previous study the localization of active contacts of pallidal electrodes in dystonia and Parkinson’s disease was not correlated with clinical outcome [9]. On the other hand, our data are difficult to reconcile with several other studies emphasizing the influence of lead location on clinical responses. Retrospective analysis of non-responders by Pauls et al. attributed most of the failed responses to misplaced leads [8]. Their conclusion, however, may be misleading as nothing is known about lead location in responders. Admittedly, the accepted range for proper lead placement, which was defined based on a commissure-based analysis was rather large in this study. Nonetheless, several active contacts of responders from our study have been found outside the permitted range, and these had to be regarded as misplaced. We hypothesize that the same had been observed in the Pauls et al. study had they refrained from excluding responders in their analysis.

In a study by Tisch et al. arm and trunk dystonia responded better to stimulation of postero-ventral portions of the GPi as opposed to more antero-dorsal regions [11]. Of note, loss of efficacy with more anterior stimulation is supported by our findings that the VTAs of more anteriorly located leads were larger. Still we could not detect a correlation between lead location and clinical responses.

Additionally, an investigation of VTAs of pallidal electrodes in *TOR1A* gene mutation carriers attributed the most pronounced clinical improvements to stimulation of the middle aspect of the posterior GPi [18]. Another study correlating theta oscillations with clinical outcome in CD patients localized the optimal spot for stimulation within the posterior third of the GPi [19]. A recent multicenter study in patients with cervical and generalized dystonia applied a probabilistic approach and suggested that the optimal spot for antidystonic effects was the ventro-posterior GPi and the adjacent subpallidal white matter [15]. In our study, the average commissure-based lead location is slightly distant from the target reported by Reich et al. with a more lateral electrode localization in the present study (*x* = 21.4 ± 1.5 mm vs. Reich et al. 19.8 ± 1.8 mm) [15]. Programming in all of our dystonia patients was not limited by pyramidal side effects and none of our patients experienced speech or gait disturbances during long-term stimulation [20].

Of note, VTA coverage of the GPi in responders was less than in intermediate/non-responders. However, a statistically significant difference was only observed for the left hemisphere. Although GPi stimulation represents the official label of the investigated DBS therapy, our data do not corroborate the concept that preferential stimulation of the GPi proper is the driver for most beneficial therapeutic effects. The largest coverage of VTAs was observed for the subpallidal area below the lateral GPi and medial GPe. This is in line with recently published results where the optimal antidystonic spot also covered the subpallidal area [15]. It is likely that the stimulation of efferent pallidothalamic fibres brings about most of the therapeutic effects observed in dystonia patients [21–23].

Limitations of the present study are its retrospective design, non-blinded dystonia ratings and the rather small and heterogeneous patient cohort. Despite these limitations the average clinical improvement in our study is well in line with previously published data from large multicenter trials in CD and GD patients [1, 4, 15]. The rate of non-responders in our cohort is also comparable to a recently published multicenter study including 105 dystonia patients [15]. Although, we did not observe a statistically significant difference in VTA size between groups, the VTAs in the left hemisphere of intermediate/non-responders were smaller. Lack of significance may be due to small sample size, and the authors cannot rule out that larger VTAs in the left hemisphere of intermediate/non-responders had resulted in additional benefit. This, however, is regarded rather unlikely as in the beginning of DBS therapy extensive programming had been performed in all non-responders including ramping up of stimulation amplitude beyond the currently used parameters.

Given the fact that there was a considerable, almost complete overlap of the VTAs of responders and intermediate/non-responders we assume that other factors apart from electrode location crucially contribute to clinical outcome after surgery. This may include genetic factors [7, 24, 25], clinical distribution of dystonia [1, 4] or demographic characteristics of the patients [5, 6, 26]. For example, shorter disease duration, lower preoperative BFMDRS scores and the presence of a *TORA1* mutation have been identified as positive outcome predictors [5–7]. In the future, additional neurophysiological investigations, e.g., the extent of abnormal sensorimotor plasticity, may be used to select patients suited for GPi stimulation [27]. Other neurophysiological markers, such as pallidal theta oscillations, might also evolve as useful guidance for optimal electrode placement [19].

## Conclusion

In the present cohort of dystonia patients VTAs were localized in ventral parts of the posterior GPi and GPe and also covered the adjacent subpallidal area, largely containing pallidothalamic outflow projections. Our data corroborate the concept that DBS of efferent pallidothalamic fibres funneling below the GPi is key to the alleviation of dystonic symptoms. However, this pattern did not differ between responders and intermediate/non-responders. In our cohort lead location did not explain lack of response in patients failing DBS therapy. Our results do not support the common notion of lead location being one of the most crucial factors for failure of pallidal deep brain stimulation. Our data rather suggest that exact lead location within the pallidum matters much less than intrinsic patient factors provided that electrodes are consistently located within a certain range within the postero-ventral pallidum.

## Electronic supplementary material

Below is the link to the electronic supplementary material.

Supplementary file1 (TIFF 1521 kb)
