# Supplementary figures and images for: Pallidal lead placement in dystonia: leads of non-responders are contained within an anatomical range defined by responders
Source: J Neurol. 2020 Feb 17;267(6):1663–71. doi: 10.1007/s00415-020-09753-z (PMC7293687; doi:10.1007/s00415-020-09753-z)

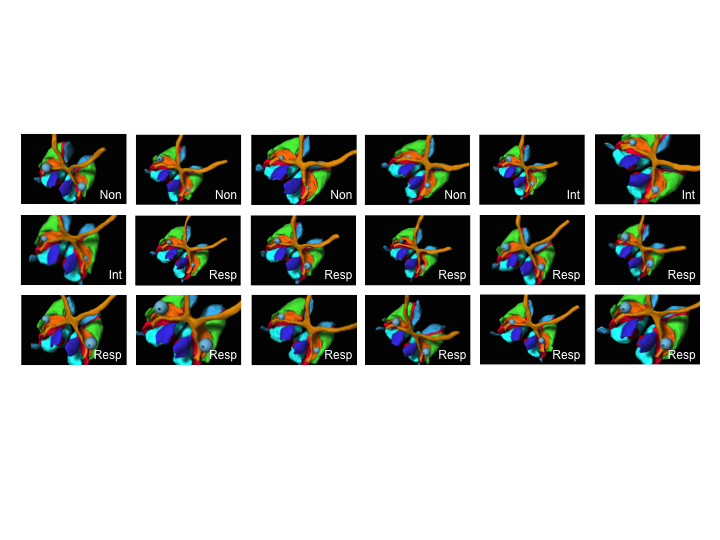

Supplement: Supplementary file 1 — Supplementary file1 (TIFF 1521 kb) [file 415_2020_9753_MOESM1_ESM.tiff]
